# Development and validation of models for predicting the overall survival and cancer-specific survival of patients with primary vaginal cancer: A population-based retrospective cohort study

**DOI:** 10.3389/fmed.2022.919150

**Published:** 2022-08-29

**Authors:** Wei-Li Zhou, Yang-Yang Yue

**Affiliations:** ^1^Department of General Surgery, Shengjing Hospital, China Medical University, Shenyang, China; ^2^Department of Health Management, Shengjing Hospital, China Medical University, Shenyang, China

**Keywords:** chemotherapy, lymphadenectomy, M stage, N stage, nomogram, radiotherapy, tumor size, vaginal cancer

## Abstract

**Background:**

No models have been developed to predict the survival probability for women with primary vaginal cancer (VC) due to VC’s extreme rareness. We aimed to develop and validate models to predict the overall survival (OS) and cancer-specific survival (CSS) of VC patients.

**Methods:**

A population-based multicenter retrospective cohort study was carried out using the 2004–2018 Surveillance, Epidemiology, and End Results Program database in the United States. The final multivariate Cox model was identified using the Brier score and Harrell’s C concordance statistic (C-statistic). The decision curve, calibration plot, and area under the time-dependent receiver operating characteristic curve (AUC) were used to evaluate model prediction performance. Multiple imputation followed by bootstrap was performed. Bootstrap validation covered the entire statistic procedure from model selection to baseline survival and coefficient calculation. Nomograms predicting OS and CSS were generated.

**Results:**

Of the 2,417 eligible patients, 1,692 and 725 were randomly allocated to the training and validation cohorts. The median age (Interquartile range) was 66 (56–78) and 65 (55–76) for the two cohorts, respectively. Our models had larger net benefits in predicting the survival of VC patients than the American Joint Committee on Cancer stage, presenting great discrimination ability and excellent agreement between the expected and observed events. The performance metrics of our models were calculated in three cohorts: the training cohort, complete cases of the validation cohort, and the imputed validation cohort. For the OS model in the three cohorts, the C-statistics were 0.761, 0.752, and 0.743. The slopes of the calibration plots were 1.017, 1.005, and 0.959. The 3- and 5-year AUCs were 0.795 and 0.810, 0.768 and 0.771, and 0.770 and 0.767, respectively. For the CSS model in the three cohorts, the C-statistics were 0.775, 0.758, and 0.755. The slopes were 1.021, 0.939, and 0.977. And the 3- and 5-year AUCs were 0.797 and 0.793, 0.786 and 0.788, and 0.757 and 0.757, respectively.

**Conclusion:**

We were the first to develop and validate exemplary survival prediction models for VC patients and generate corresponding nomograms that allow for individualized survival prediction and could assist clinicians in performing risk-adapted follow-up and treatment.

## Introduction

Primary vaginal cancer (VC) is a rare gynecologic cancer, accounting for 2% of all gynecologic cancer cases, with about 18,000 new cases and 8,000 deaths worldwide in 2020 (1). The VC incidence between 1999 and 2015 remained stable among women aged 40–69 (2). Histologically, vaginal carcinoma mainly includes squamous cell carcinomas (SCC, accounting for 80–90%) and adenocarcinomas (ADE, 4–10%) (3, 4). SCC and ADE present a similar etiology and prognosis; hence they are routinely treated with the same therapy methods (5, 6). Currently, the primary treatment for VC is surgical resection combined with radiotherapy with or without chemotherapy (7–11).

Age, tumor size, lymph node invasion, distant metastasis, radiotherapy, chemotherapy, and surgery type are significant prognostic survival factors for VC patients (7, 9, 11–14). However, no studies have integrated those variables into a single model. Moreover, the staging systems that reflect the severity and extension of VC include the American Joint Committee on Cancer (AJCC) stage (15, 16), the TNM stage of AJCC, and the International Federation of Gynaecology and Obstetrics (FIGO) stage (17). The FIGO stage is a system commonly used by gynecological clinicians, which can be derived from the AJCC stage (4, 17–19). Thus, the AJCC and TNM stages should be estimated during model development and validation.

Lymphadenectomy and sentinel lymph nodes biopsy (SLNB) are two techniques to detect lymph node status. Lymphadenectomy has a higher complication occurrence rate due to its aggression. SLNB is helpful in patients undergoing surgery because lymphatic drainage from the primary lesion does not always follow the anatomically lymphatic channels that would have been predicted (20, 21). However, SLNB challenges the surgical techniques of healthcare centers due to its inherent complexity. Lymphadenectomy and SLNB should be considered during model development.

Nomograms for predicting the overall survival (OS) and cancer-specific survival (CSS) of vulvar cancer patients have been well developed, with a Harrell’s C concordance index (C-statistic) of 0.83–0.85 (22–25). However, no nomograms have been developed to predict the OS or CSS for VC patients due to VC’s extreme rareness, which causes the unfeasibility of developing prediction models, especially within a single healthcare center. Given nomograms’ significant clinical practice value, it is essential to generate nomograms predicting the survival of VC patients. The increased cases in the population-based database made it possible to get adequate VC cases to develop nomograms.

Hence, we aimed to develop and validate nomograms that predict the OS and CSS of VC patients using a population-based multicenter database.

## Materials and methods

We carried out the study following the Transparent Reporting of a multivariable prediction model for Individual Prognosis Or Diagnosis (TRIPOD) guideline for prognostic models (26). An ethical review and informed consent were waived for the study because we used de-identified publicly available data obtained from the Surveillance, Epidemiology, and End Results (SEER) Program database of the National Cancer Institute (27). We have signed the Data-use Agreement for the SEER 1975–2018 Research Data File.

### Study population

Patients affected by C52.9 VC (as classified by the International Classification of Diseases for Oncology, 3rd Edition codes) diagnosed between January 01, 2004 and December 31, 2018 were selected from the sub-database of the SEER database (the Incidence-SEER Research Plus Data, 18 registries, Nov 2020 sub [2000–2018]) (27). Patients whose VC was not SCC or ADE, not confirmed by positive histology, or not the first tumor were excluded. Those under 18 or over 100 years or with T0 stage VC were excluded.

### Variables and outcomes

The variables assessed in this study included the year of diagnosis, age, marital status, race, tumor size, pathological grade, histology type, radiotherapy, chemotherapy, number of lymph nodes removed, SLNB, surgery type, the presence of other malignancies, the AJCC stage, T, N, and M stages. Those variables were derived from the corresponding data fields of the SEER database. Surgery types were categorized into four groups: none, local tumor excision (LTE), vaginectomy, and debulking. LTE included electrocautery, fulguration (includes hot forceps for tumor destruction), laser, local tumor excision-not otherwise specified (NOS), photodynamic therapy, electrocautery, cryosurgery, laser ablation, laser excision, polypectomy, and excisional biopsy. Vaginectomy included simple or partial surgical removal of the primary site, total surgical removal of the primary site, enucleation, radical surgery, and surgery-NOS. A 0-month survival time was recorded as 0.5 to more accurately represent cases that survived less than 1 month from their diagnosis (28).

The primary outcomes were OS and CSS. OS was defined as the period from the date of diagnosis to the date of death for any reason; alive patients were censored. CSS was defined as the period from the date of diagnosis to the date of death for the reason of VC, while alive patients and those not dead of VC were censored.

### Model developing procedure and statistical analysis

A two-tailed *p*-value < 0.05 was considered statistically significant. The statistical processes were performed using the STATA 17.0 software (StataCorp, College Station, TX, United States). The data was analyzed from September 01, 2021 to March 20, 2022.

The final samples were randomly split into the training and internal validation cohorts using a ratio of 7:3 (1,692 vs. 725 patients), with the constraints of keeping the proportion of outcome events balanced between the two cohorts according to the TRIPOD guideline (26). We used the Chi-square test to investigate the balance of variables between the two cohorts. The Kruskal–Wallis *H* test was used to compare the between-cohort difference in age and follow-up time.

Multiple imputation using a chained equation with 10 imputed samples was carried out to impute surgery type, number of lymph nodes removed, marital status, pathological grade, and tumor size. The independent variables used during multiple imputations included the year of diagnosis, age, race, radiotherapy, chemotherapy, pathological grade, histology type, presence of other malignancies, and T, N, and M stages. The SLNB variable was neither imputed nor included as a predictor to impute other variables because 92.8% of final samples did not receive SLNB. The imputation procedures were performed separately in the training and validation cohorts to prevent information leakage from each other (29). The sufficiency of the number of imputations was assessed using the fraction of missing information. In the study, ten imputations were enough. Then bootstrap with replacement using 200 repetitions was performed to calculate the Brier score and C-statistic to assess the performance of candidate models. A larger value of the Brier score and C-statistic indicates a better prediction performance of a model. Only if a candidate model with an indicator at least 3% greater than others was considered a better model. If two models had similar indicators, the one including fewer variables was selected as the better model. After a comparison of all candidate models, the final models were identified.

Several nested candidate multivariate Cox models were evaluated in this study. OS candidate models were generated by dropping one insignificant variable with small beta coefficients from a previous model once a time (see Supplementary Tables 1, 2). CSS candidate models were generated similarly, with all the variables in the final OS model kept in CSS models, no matter whether those variables were statistically significant.

The best fit models were refitted on the imputed training cohort using bootstrap with 200 repetitions to calculate the imputation-averaged 3-, 5-year baseline survival and the imputation-averaged coefficients with standard errors.

Next, based on previously calculated baseline survivals and coefficients, the patient-level probabilities of death were calculated within the imputed training cohort, the complete cases of the validation cohort, and the imputed validation cohort. According to the definition of the Cox proportional hazard model (30), the probabilities can be calculated as follows (see Supplementary Document for details):


Theprobabilityofthe 3-yearOS=S0,OS(3)exp(XBOS)=0.82548exp(XBOS)×100%

Theprobabilityofthe 5-yearOS=S0,OS(5)exp(XBOS)=0.74510exp(XBOS)×100%

Theprobabilityofthe 3-yearCSS=S0,CSS(3)exp(XBCSS)=0.84248exp(XBCSS)×100%

Theprobabilityofthe 5-yearCSS=S0,CSS(5)exp(XBCSS)=0.78676exp(XBCSS)×100%

Within the imputed training and validation cohorts, the patient-level probability of death was a single value calculated by averaging the failure probabilities for the patient in each of the ten imputed samples. Furthermore, the decision curve, calibration plot, and time-dependent receiver operating characteristic (ROC) curve were plotted based on the previously calculated probability of death to assess the final model’s prediction performance (31). The 95% confidence intervals (95% CIs) of the slope of the calibration plot, C-statistic, and AUC of the time-dependent ROC were calculated using bootstrap with 200 repetitions.

Based on the final models, nomograms for predicting 3- and 5-year OS and CSS were generated using a modified “nomocox” command based on pre-calculated baseline survivals (32).

## Results

### Baseline characteristics

**FIGURE 1:**
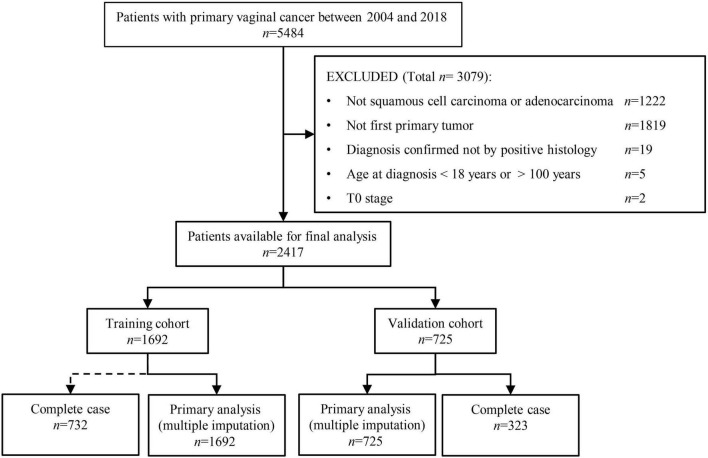
Patient selection procedure. Of the 5484 vaginal cancer patients diagnosed between 2004 and 2018, 1,692 and 725 patients were finally selected into the training and validation cohorts, respectively.

Of the 2,417 patients selected in this study, 1,692 (70%) and 725 (30%) were randomly allocated to the training and internal validation cohorts (see Figure 1). The median age (Interquartile range) was 66 (56–78) and 65 (55–76) for patients in the training and validation cohorts, respectively. The year of diagnosis, age at diagnosis, marital status, race, tumor size, pathological grade, radiotherapy, chemotherapy, histology type, number of lymph nodes removed, SLNB, surgery type, AJCC stage, T, N, M stages, and the presence of other tumors were balanced between the training and validation cohorts (Chi-square *p* > 0.05 for all), except for the slightly more patients undergoing chemotherapy in the validation cohort (55.9% vs. 51.4%, *p* = 0.04). There was no difference in the proportion of outcome events between the two cohorts (*p* > 0.05). The two cohorts had comparable follow-up time (25.5 months [interquartile range 9–68.5] vs. 28 months [9–76], *p* = 0.651, see Table 1).

**TABLE 1:** Composition proportion of each baseline characteristic in the training and validation cohorts derived from the final selected vaginal cancer patients diagnosed between 2004 and 2018.

Characteristics	Training	Validation	*p*-value
	cohort	cohort	
	No. (%)	No. (%)	
**Year of diagnosis**			0.563
2004–2009	644(38.1)	285(39.3)	
2010–2018	1048(61.9)	440(60.7)	
**Age, median (IQR), y**	66(56–78)	65(55–76)	0.188
**Age, y**			0.181
18–39	58(3.4)	29(4.0)	
40–59	522(30.9)	237(32.7)	
60–79	726(42.9)	322(44.4)	
80–100	386(22.8)	137(18.9)	
**Marital status**			0.266
Married	599(35.4)	272(37.5)	
Single	289(17.1)	122(16.8)	
Divorced/widowed/separated	661(39.1)	286(39.4)	
Missing	143(8.5)	45(6.2)	
**Race**			0.459
White	1310(77.4)	572(78.9)	
Black	253(15.0)	108(14.9)	
Other	129(7.6)	45(6.2)	
**Tumor size, cm**			0.069
<2	138(8.2)	74(10.2)	
2–4	361(21.3)	132(18.2)	
≥4	568(33.6)	267(36.8)	
Missing	625(36.9)	252(34.8)	
**Pathological grade**			0.929
Well	143(8.5)	57(7.9)	
Moderately	491(29.0)	207(28.6)	
Poorly/undifferentiated	518(30.6)	230(31.7)	
Missing	540(31.9)	231(31.9)	
**Histology type**			0.436
Squamous cell carcinoma	1397(82.6)	589(81.2)	
Adenocarcinoma	295(17.4)	136(18.8)	
**Radiotherapy**			0.902
None/unknown	437(25.8)	200(27.6)	
Beam	689(40.7)	283(39.0)	
Beam plus implants	436(25.8)	188(25.9)	
Radiation, NOS	41(2.4)	18(2.5)	
Implants	89(5.3)	36(5.0)	
**Chemotherapy**			0.042
None/unknown	823(48.6)	320(44.1)	
Yes	869(51.4)	405(55.9)	
**Number of lymph nodes removed**			0.175
None	1473(87.1)	604(83.3)	
1–3	33(2.0)	21(2.9)	
≥4	150(8.9)	80(11.0)	
Unknown number	14(0.8)	7(1.0)	
Missing	22(1.3)	13(1.8)	
**Sentinel lymph nodes biopsy**			0.369
No	1662(98.2)	706(97.4)	
Yes	8(0.5)	6(0.8)	
Missing	22(1.3)	13(1.8)	
**Surgery**			0.113
None	1182(69.9)	520(71.7)	
Local tumor excision	222(13.1)	74(10.2)	
Vaginectomy	267(15.8)	118(16.3)	
Debulking	12(0.7)	4(0.6)	
Missing	9(0.5)	9(1.2)	
**Other malignancies**			0.296
No	1487(87.9)	626(86.3)	
Yes	205(12.1)	99(13.7)	
**AJCC stage**			0.686
I	474(28.0)	195(26.9)	
II	399(23.6)	176(24.3)	
III	303(17.9)	151(20.8)	
IV	2(0.1)	1(0.1)	
IVA	120(7.1)	44(6.1)	
IVB	214(12.6)	86(11.9)	
Missing	180(10.6)	72(9.9)	
**T stage**			0.594
T1	594(35.1)	250(34.5)	
T2	526(31.1)	229(31.6)	
T3	224(13.2)	110(15.2)	
T4	184(10.9)	67(9.2)	
TX	164(9.7)	69(9.5)	
**N stage**			0.703
N0	1185(70.0)	497(68.6)	
N1	321(19.0)	148(20.4)	
NX	186(11.0)	80(11.0)	
**M stage**			0.473
M0	1417(83.7)	619(85.4)	
M1	214(12.6)	86(11.9)	
MX	61(3.6)	20(2.8)	
**Follow-up time, median (IQR), mo**	25.5(9–68.5)	28(9–76)	0.651
**Outcome**			0.999
Alive	796(47.0)	341(47.0)	
Dead of vaginal cancer	646(38.2)	277(38.2)	
Dead of other reasons	237(14.0)	101(13.9)	
Dead of unknown reason	13(0.8)	6(0.8)	

IQR, interquartile range; NOS, not otherwise specified; AJCC, the American Joint Committee on Cancer.

### Candidate model selection

In order to select the final models, the model performance indicators of candidate models were calculated using bootstraps with 200 repetitions within the imputed training cohort. The prediction performance of the candidate models is summarized in Table 2. The final models (Model 5 for both OS and CSS) were selected because they had the least number of variables but similar performance to other candidate models (within a 3% difference in indicator values). The final OS model included age, tumor size, radiotherapy, chemotherapy, surgery, number of lymph nodes removed, and T, N, and M stages. The final CSS model included the same variables and an indicator variable of the presence of other malignancies.

**TABLE 2:** Brier scores and C-statistics of candidate multivariate Cox proportional hazard models in predicting patient overall survival and cancer-specific survival outcome within the imputed training cohort.

Indicators	Overall survival		Cancer-specific survival	
	Value	95% confidence interval	Value	95% confidence interval
**Brier score**				
Model 1	0.163	0.133–0.192	0.138	0.110–0.166
Model 2	0.287	0.258–0.317	0.241	0.213–0.269
Model 3	0.288	0.259–0.317	0.244	0.216–0.272
Model 4	0.291	0.261–0.321	0.244	0.216–0.272
Model 5	0.290	0.261–0.319	0.243	0.215–0.272
**C-statistic**				
Model 1	0.839	0.812–0.865	0.853	0.825–0.882
Model 2	0.847	0.820–0.874	0.857	0.828–0.886
Model 3	0.846	0.820–0.873	0.858	0.829–0.887
Model 4	0.848	0.822–0.874	0.855	0.826–0.884
Model 5	0.844	0.817–0.871	0.855	0.826–0.883

Model 5 is the final selected model; See more details in the Supplementary Tables 1, 2.

### Results of the final models

The results of the final multivariate Cox proportional hazard models for predicting the OS and CSS are shown in Table 3. As presented in the table, age, tumor size, radiotherapy, chemotherapy, number of lymph nodes removed, and T, N, and M stages were all significantly associated with OS (*p* < 0.001). However, the association of chemotherapy, number of lymph nodes removed, and N stage with CSS were insignificant. Additionally, the presence of other malignancies was significantly correlated with CSS (*p* < 0.001).

**TABLE 3:** Beta coefficients and their bootstrap standard errors of the final overall and cancer-specific survival models calculated within the imputed training cohort.

Variables	Overall survival			Cancer-specific survival		
	β coefficients	Bootstrap *SE*	*p*-value	β coefficients	Bootstrap *SE*	*p*-value
**Age, y**						
18–39	Reference			Reference		
40–59	0.19593	0.27779	0.48060	0.06896	0.28024	0.80564
60–79	0.67730	0.26537	0.01070	0.35783	0.27253	0.18918
80–100	1.46783	0.27607	<0.00001	1.09818	0.28078	0.00009
**Tumor size, cm**						
<2	Reference			Reference		
2–4	0.21352	0.14344	0.13660	0.24540	0.17694	0.16548
≥4	0.44749	0.13988	0.00138	0.57388	0.18809	0.00228
**Radiotherapy**						
None	Reference			Reference		
Beam	−0.43073	0.09575	0.00001	−0.48371	0.12296	0.00008
Beam + implants	−0.86705	0.11851	<0.00001	−1.04213	0.15922	<0.00001
Radiation, NOS	−0.29244	0.20267	0.14904	−0.23287	0.23471	0.32112
Implants	−0.95970	0.18768	<0.00001	−1.13098	0.23740	<0.00001
**Chemotherapy**						
None/unknown	Reference			Reference		
Yes	−0.27861	0.08434	0.00096	−0.19033	0.10500	0.06989
**Surgery**						
None	Reference			Reference		
Local tumor excision	−0.54175	0.11260	<0.00001	−0.61139	0.15755	0.0001
Vaginectomy	−0.73283	0.14495	<0.00001	−0.67324	0.17219	0.00009
Debulking	−0.26163	0.36299	0.47105	−0.07539	0.37202	0.83941
**Number of lymph nodes removed**						
None	Reference			Reference		
1–3	0.17467	0.30427	0.56593	0.17230	0.38622	0.65551
≥4	−0.47991	0.19393	0.01334	−0.36552	0.21715	0.09233
Number unknown	0.38244	0.40080	0.33999	0.67311	3.21673	0.83425
**T stage**						
T1	Reference			Reference		
T2	0.23081	0.09139	0.01155	0.36817	0.12840	0.00414
T3	0.41905	0.12574	0.00086	0.61803	0.14603	0.00002
T4	0.82441	0.14587	<0.00001	0.99892	0.16405	<0.00001
TX	0.12220	0.17625	0.48809	0.27493	0.19949	0.16815
**N stage**						
N0	Reference			Reference		
N1	0.26231	0.10441	0.01200	0.23417	0.12536	0.06176
NX	0.25829	0.14786	0.08066	0.06867	0.19146	0.71986
**M stage**						
M0	Reference			Reference		
M1	0.66769	0.10629	<0.00001	0.71244	0.13504	<0.00001
MX	−0.20732	0.24499	0.39743	0.10063	0.26910	0.70845
**Presence of other malignancies**						
No	–			Reference		
Yes	–	–	–	−0.49258	0.14590	0.00074
**Baseline survival**						
3 years	0.82548	–	–	0.84248	–	–
5 years	0.74510	–	–	0.78676	–	–

SE, standard error; NOS, not otherwise specified; see Supplementary Document for details about the calculation of patient-level survival probability.

### Model prediction performance

**FIGURE 2:**
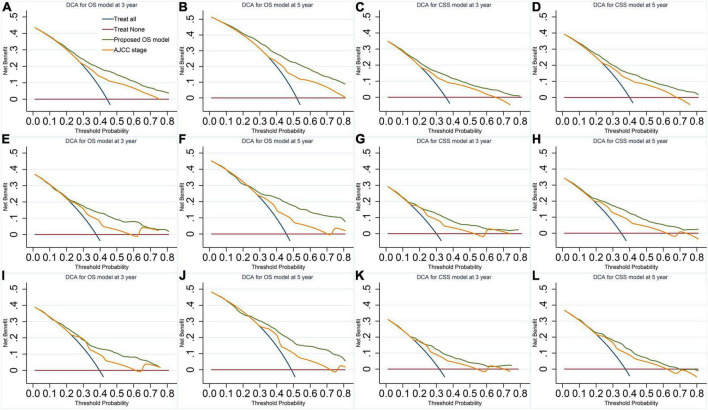
Decision curves of the final overall survival (OS) and cancer-specific survival (CSS) models. **(A,B)** OS model in the imputed training cohort. **(C,D)** CSS model in the imputed training cohort. **(E,F)** OS model in the complete cases of the validation cohort. **(G,H)** CSS model in the complete cases of the validation cohort. **(I,J)** OS model in the imputed validation cohort. **(K,L)** CSS model in the imputed validation cohort. The plots of decision curves illustrate that our models have larger net benefits than the American Joint Committee on Cancer stage in predicting the 3- and 5-year survival of vaginal cancer patients, showing better clinical usefulness.

**FIGURE 3:**
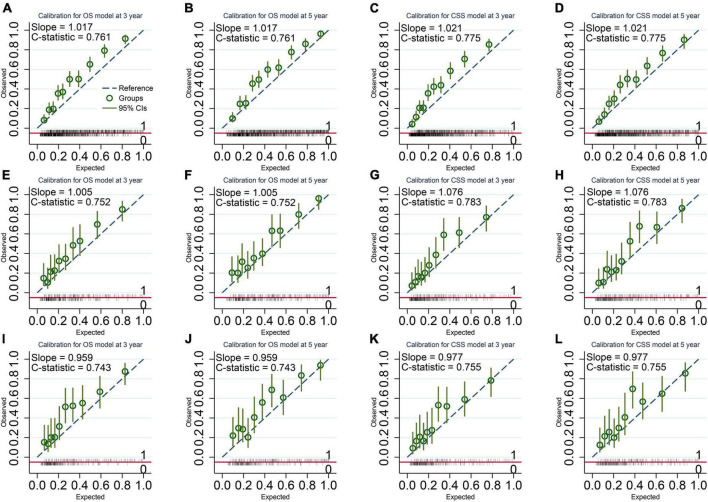
Calibration Plots of the final overall survival (OS) and cancer-specific survival (CSS) models. **(A,B)** OS model in the imputed training cohort with a C-statistic of 0.761 and a slope of 1.017. **(C,D)** CSS model in the imputed training cohort with a C-statistic of 0.775 and a slope of 1.021. **(E,F)** OS model in the complete cases of the validation cohort with a C-statistic of 0.752 and a slope of 1.005. **(G,H)** CSS model in the complete cases of the validation cohort with a C-statistic of 0.783 and a slope of 1.076. **(I,J)** OS model in the imputed validation cohort with a C-statistic of 0.743 and a slope of 0.959. **(K,L)** CSS model in the imputed validation cohort with a C-statistic of 0.755 and a slope of 0.977. The calibration plots show a good agreement between the expected outcome predicted by our model and the observed outcome.

**FIGURE 4:**
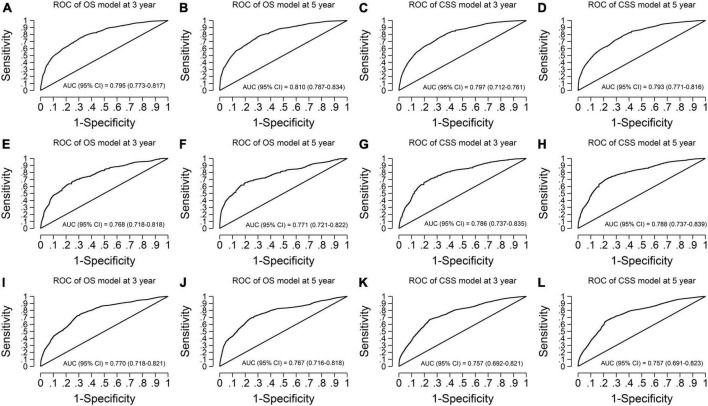
Time-dependent receiver operating characteristic curves of the final overall survival (OS) and cancer-specific survival (CSS) models. **(A,B)** OS model in the imputed training cohort with AUCs of 0.795 and 0.810 at 3- and 5-year survival time, respectively. **(C,D)** CSS model in the imputed training cohort with AUCs of 0.797 and 0.793 at 3- and 5-year survival time, respectively. **(E,F)** OS model in the complete cases of the validation cohort with AUCs of 0.768 and 0.771 at 3- and 5-year survival time, respectively. **(G,H)** CSS model in the complete cases of the validation cohort with AUCs of 0.786 and 0.788 at 3- and 5-year survival time, respectively. **(I,J)** OS model in the imputed validation cohort with AUCs of 0.770 and 0.767 at 3- and 5-year survival time, respectively. **(K,L)** CSS model in the imputed validation cohort with AUCs of 0.757 and 0.757 at 3- and 5-year survival time, respectively. The receiver operating characteristic curves show our models have good discrimination ability in predicting the 3- and 5-year survival of vaginal cancer patients. AUC, area under the receiver operating characteristic curve.

Our models have more considerable net benefits than the AJCC stage, showing excellent clinical practice usefulness (Figure 2). Moreover, the calibration plots show a good agreement between the expected and observed events (Figure 3). The time-dependent ROC curves are displayed in Figure 4.

For the OS model, the C-statistics were 0.761, 0.752, and 0.743 in the imputed training cohort, the complete cases of the validation cohort, and the imputed validation cohort, respectively. The slopes of the calibration plots were 1.017, 1.005, and 0.959 in the three cohorts. The 3-year AUCs were 0.795, 0.768, and 0.770. The 5-year AUCs were 0.810, 0.771, and 0.767 (Table 4).

**TABLE 4:** Performance metrics of the final overall survival and cancer-specific survival models in predicting patient survival outcome within the imputed training cohort, the complete cases of the validation cohort, and the imputed validation cohort.

Performance	Training cohort (imputed)	Validation cohort (complete cases)	Validation cohort (imputed)
**Overall survival**			
C-statistic	0.761 (0.745–0.777)	0.752 (0.717–0.787)	0.743 (0.706–0.779)
Calibration slope	1.017 (0.942–1.092)	1.005 (0.848–1.162)	0.959 (0.777–1.141)
3-year AUC	0.795 (0.773–0.817)	0.768 (0.718–0.818)	0.770 (0.718–0.821)
5-year AUC	0.810 (0.787–0.834)	0.771 (0.721–0.822)	0.767 (0.716–0.818)
**Cancer-specific survival**			
C-statistic	0.775 (0.759–0.791)	0.758 (0.723–0.793)	0.755 (0.710–0.800)
Calibration slope	1.021 (0.931–1.111)	0.939 (0.769–1.109)	0.977 (0.755–1.199)
3-year AUC	0.797 (0.712–0.761)	0.786 (0.737–0.835)	0.757 (0.692–0.821)
5-year AUC	0.793 (0.771–0.816)	0.788 (0.737–0.839)	0.757 (0.691–0.823)

AUC, the area under the receiver operating characteristic curve. Numbers in parentheses are the bootstrapped 95% confidence interval.

For the CSS model, the C-statistics were 0.775, 0.758, and 0.755 in the three cohorts. The slopes of the calibration plots were 1.021, 0.939, and 0.977. The 3-year AUCs were 0.797, 0.786, and 0.757. The 5-year AUCs were 0.793, 0.788, and 0.757 (Table 4).

### Nomograms for predicting the 3- and 5-year survival

**FIGURE 5:**
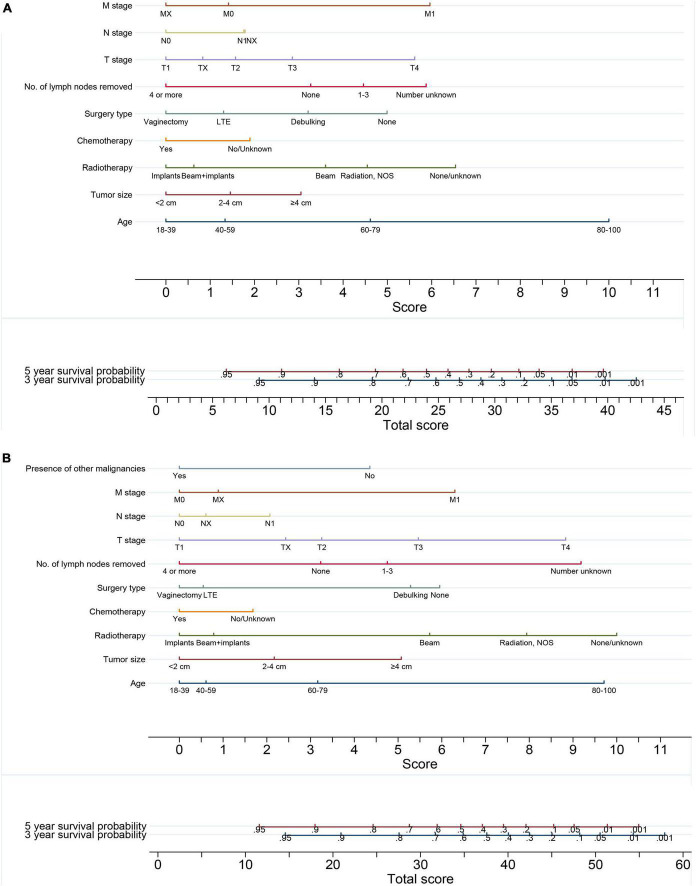
Nomograms for predicting the 3- and 5-year **(A)** overall survival and **(B)** cancer-specific survival of vaginal cancer patients. By drawing a vertical line straight down to the horizontal axis labeled with points and summing every single score of each factor, the probabilities of 3- and 5-year survival were the probabilities corresponding to the total scores.

The baseline survivals and coefficients of the final models calculated on the imputed training cohort were used to generate the nomograms for predicting the probability of 3- and 5-year OS (Figure 5A) and CSS (Figure 5B) for VC patients. By drawing a vertical line straight down to the horizontal axis labeled with points and summing every single score of each factor, the patient’s probabilities of 3- or 5-year survival were the probabilities corresponding to the total scores.

## Discussion

This retrospective cohort study developed and validated models for predicting the 3-and 5-year OS and CSS for VC patients based on a cohort of 2,417 cases from a population-based multicenter database. Our models with superb discrimination and calibration have a more considerable net benefit than the AJCC stage, showing excellent clinical usefulness. Using the corresponding nomograms, which provided a convenient and well-calibrated survival prediction tool, clinicians could calculate patient-level prognostication of survival, recommend intensive clinical follow-up for high-risk patients, and perform the risk-adapted treatment.

The variables included in our models involved age at diagnosis, tumor size, radiotherapy type, chemotherapy, surgery type, number of lymph nodes removed, T stage, N stage, M stage, and the presence of other malignancies. Those variables were regularly inspected characteristics in clinical practice. To our knowledge, no models integrating those factors have been developed to predict the survival of VC patients due to the VC’s extreme rareness (33, 34). We are the first to integrate those factors into a single survival prediction model and build nomograms predicting VC patients’ survival using a large representative population-based cohort. The OS and CSS models contained the same variables, except for the presence of other malignancies for CSS. Accordingly, the probabilities of OS and CSS can be determined simultaneously, intensifying our models’ practical usefulness. Another unique characteristic is that we bootstrapped the entire modeling process, including model selection, performance indicator generation, baseline survival, coefficient, and standard error calculation, which further enhanced the generalizability of our models. Besides, we assessed the internal validity with bootstrap for a more realistic estimate of the prediction performance of the models in similar future patients.

Another strength of this study is that multiple imputation was used to generate 10 sets of imputed samples, which increased the usable sample size and made the calculated coefficients closer to the actual value and their standard error range narrower. Multiple imputation could reduce the complete case biases caused by the poor representation of the complete case. Internal validation was performed in the complete case of the validation cohort and the imputed validation cohort, showing similar results, which further confirmed the excellent performance of our models (35, 36). Moreover, multiple imputation were followed by the bootstrap technique in this study, which made our models capture much more uncertainty and increased their generalizability.

This study confirmed that older age and larger tumor size were negatively associated with survival, consistent with other studies (4, 11, 12, 37–39). We also found that a higher tumor stage was negatively correlated with the survival of VC patients, similar to previous studies (4, 17, 38–40). Instead of using a single FIGO or AJCC stage, we investigated T, N, and M stages in our models because they show a more elaborate representation of tumor progress than a single stage. The significant association of the N stage with survival agreed with published studies that also found a correlation between lymph node invasion and survival (12, 40). To further investigate the effect of lymph node resection, we also controlled the number of lymph nodes removed and found its significant association with survival. The number of lymph nodes removed in the models also contributed to a more precise survival prediction, adapting to modern surgical technique development.

Moreover, we discovered that radiotherapy, chemotherapy, and more aggressive surgery were positively correlated with survival, in agreement with other studies (9, 11, 12, 14, 38–41). Surgery combined with radiotherapy and chemotherapy is still the primary treatment for VC (42). The radiotherapy in the SEER database is classified into beam radiation, radioactive implants, radioisotopes, beam plus implants (combination of beam radiation with radioactive implants or radioisotopes), and radiation-NOS. The radioactive implants and radioisotopes correspond to brachytherapy. We also found that beam plus implants or only implants had better effectiveness than beam radiation, similar to published studies (7, 9, 12, 14, 38, 43, 44). Some studies argued that image-guided brachytherapy might improve the effectiveness of brachytherapy (45–47). Furthermore, we found that the presence of other malignancies was a favorable prognostic factor for CSS but not for OS. That may be because a longer survival time tends to make VC patients experience an increased probability of occurring other malignancies; thus, the death due to VC was competed by other malignancies.

Additionally, we found no improvement in model prediction performance with the addition of marital status, race, pathology grade, and histology type. The lack of performance improvement reflects that significant variables embodied the effects of those variables. The insignificance of histology type reflected the similar prognostic outcome of SCC and ADE for VC.

Attention should be taken when applying those nomograms in clinical practice. We only included VC patients with SCC and ADE in the study. Accordingly, the proposed nomograms should only be reasonably used for the two histology types. Applying the nomograms to other histology types might be problematic. In addition, the nomograms were built based on patients aged 18–100. Hence, expanded application to younger or older patients should be cautious. Additionally, given that the SEER database only includes the United States population, care should be taken when those models are used on a population of other countries.

Some limitations in this study should be clarified. First, we could not control the tumor’s detailed location (the upper or lower of the vagina) because the location is unavailable in the SEER database. An upper third location is associated with more prolonged survival, maybe due to a different lymph drainage pattern (11). Although we controlled the T, N, and M stages, which could account for some effect of tumor location, there may still be confounding effects of the location. Second, due to the retrospective study’s nature, there might be missing factors highly correlated with the survival of VC patients, although we have assessed available variables suggested by previous studies. Third, external validation on a distinct population was not carried out because a sufficiently large sample from a different population was unavailable in a single healthcare center due to VC’s extreme rareness. Finally, human papillomavirus (HPV) has been argued to be positively associated with the survival of VC patients (48). However, the HPV status of VC patients was not available in the SEER database, so it could not be controlled.

## Conclusion

For predicting the probability of 3-and 5-year OS and CSS for VC patients with SCC or ADE, we developed and validated the first models and generated the first nomograms based on the models. Our models and the corresponding nomograms with excellent survival prediction performance could help clinicians perform risk-adapted follow-up and treatment on VC patients. Further prospective studies investigating more factors, such as the tumor’s location, are warranted to confirm our study’s findings and improve the prediction accuracy.

## Data availability statement

Publicly available datasets were analyzed in this study. This data can be found here: http://seer.cancer.gov.

## Ethics statement

Ethical review and approval was not required for the study on human participants in accordance with the local legislation and institutional requirements. Written informed consent for participation was not required for this study in accordance with the national legislation and the institutional requirements.

## Author contributions

W-LZ and Y-YY had full access to all the data in the study and took responsibility for the integrity of the data and the accuracy of the data analysis, organized the original individual studies concept and design, analyzed and interpreted the data, and revised the manuscript. Y-YY acquired the raw data. W-LZ drafted the manuscript. Both authors read and approved the final manuscript.
