# Supplementary material for: Development and validation of models for predicting the overall survival and cancer-specific survival of patients with primary vaginal cancer: A population-based retrospective cohort study
Source: Front Med (Lausanne). 2022 Aug 29;9:919150. doi: 10.3389/fmed.2022.919150 (PMC9464817; doi:10.3389/fmed.2022.919150)
Supplement: Supplementary file 1 [file Data_Sheet_1.docx]

**Supplement Document**

**Calculation of 3- and 5-year patient-level overall survival and cancer-specific survival probabilities**

The probability of the 3-year OS=${S_{0, OS}(3)}^{exp({XB}_{OS})}$=${0.82548}^{exp({XB}_{OS})}$× 100%

The probability of the 5-year OS=${S_{0, OS}(5)}^{exp({XB}_{OS})}$=${0.74510}^{exp({XB}_{OS})}$ × 100%

$S_{0, OS}(3)$=0.82548 (3-year baseline overall survival);

$S_{0, OS}(5)$=0.74510 (5-year baseline overall survival);

${XB}_{OS}$=0.19593 × Age (40-59) + 0.67730 × Age (60-79) + 1.46783 × Age (80-100) + 0.21352 × Tumor size (2-4) + 0.44749 × Tumor size (≥4) - 0.43073 × Radiotherapy (Beam) - 0.86705 × Radiotherapy (Beam plus implants) - 0.29244 × Radiotherapy (Radiation-NOS) - 0.95970 × Radiotherapy (Implants) - 0.27861 × Chemotherapy (Yes) - 0.54175 × Surgery (Local tumor excision) - 0.73283 × Surgery (Vaginectomy) -0.26163 × Surgery (Debulking) + 0.17467 × Lymph nodes removed (1-3) - 0.47991 × Lymph nodes removed (≥4) + 0.38244 × Lymph nodes removed (Unknown number) + 0.23081 × T (T2)+ 0.41905 × T (T3) + 0.82441 × T (T4) + 0.12220 × T (Tx) + 0.26231 × N (N1)+ 0.25829 × N (Nx) + 0.66769 × M (M1) - 0.20732 × M (Mx);

The probability of the 3-year CSS=${S_{0, CSS}(3)}^{exp({XB}_{CSS})}$=${0.84248}^{exp({XB}_{CSS})}$ × 100%

The probability of the 5-year CSS=${S_{0, CSS}(5)}^{exp({XB}_{CSS})}$=${0.78676}^{exp({XB}_{CSS})}$ × 100%

$S_{0, CSS}(3)$=0.84248 (3 year baseline cancer-specific survival);

$S_{0, CSS}(5)$=0.78676 (5 year baseline cancer-specific survival);

${XB}_{CSS}$= 0.06896 × Age (40-59) + 0.35783 × Age (60-79) + 1.09818 × Age (80-100) + 0.24540 × Tumor size (2-4) + 0.57388 × Tumor size (≥4) - 0.48371 × Radiotherapy (Beam) - 1.04213 × Radiotherapy (Beam plus implants) - 0.23287 × Radiotherapy (Radiation-NOS) - 1.13098 × Radiotherapy (Implants) - 0.19033 × Chemotherapy - 0.61139 × Surgery (Local tumor excision) - 0.67324 × Surgery (Vaginectomy) - 0.07539 × Surgery (Debulking) + 0.17230 × Lymph nodes removed (1-3) - 0.36552 × Lymph nodes removed (≥4) + 0.67311 × Lymph nodes removed (Unknown number) + 0.36817 × T (T2)+ 0.61803 × T (T3) + 0.99892 × T (T4) + 0.27493 × T (Tx) + 0.23417 × N (N1)+ 0.06867 × N (Nx) + 0.71244 × M (M1) + 0.10063 × M (Mx) - 0.49258 × other malignancies (Yes);
