# Supplementary material for: Development and validation of models for predicting the overall survival and cancer-specific survival of patients with primary vaginal cancer: A population-based retrospective cohort study
Source: Front Med (Lausanne). 2022 Aug 29;9:919150. doi: 10.3389/fmed.2022.919150 (PMC9464817; doi:10.3389/fmed.2022.919150)
Supplement: Supplementary file 2 [file Table_1.DOCX]

**Supplement Table 1| Results of the Candidate Multivariate Cox Proportional Hazard Models of Overall Survival**

| **Variables** | **Model 1** | | | **Model 2** | | | **Model 3** | | | **Model 4** | | | **Model 5** | | |
| --- | --- | --- | --- | --- | --- | --- | --- | --- | --- | --- | --- | --- | --- | --- | --- |
|  | **β** | **SE** | **p-value** | **β** | **SE** | **p-value** | **β** | **SE** | **p-value** | **β** | **SE** | **p-value** | **β** | **SE** | **p-value** |
| **Age** |  |  |  |  |  |  |  |  |  |  |  |  |  |  |  |
| 18-39 | reference |  |  | reference |  |  | reference |  |  | reference |  |  | reference |  |  |
| 40-59 | 0.199 | 0.253 | 0.430 | 0.200 | 0.253 | 0.429 | 0.207 | 0.252 | 0.413 | 0.195 | 0.251 | 0.438 | 0.206 | 0.250 | 0.410 |
| 60-79 | 0.659 | 0.249 | 0.008 | 0.658 | 0.249 | 0.008 | 0.661 | 0.249 | 0.008 | 0.653 | 0.248 | 0.009 | 0.680 | 0.245 | 0.005 |
| 80-100 | 1.427 | 0.258 | <0.001 | 1.427 | 0.258 | <0.001 | 1.438 | 0.258 | <0.001 | 1.430 | 0.257 | <0.001 | 1.475 | 0.251 | <0.001 |
| **Marital status** |  |  |  |  |  |  |  |  |  |  |  |  |  |  |  |
| Married | reference |  |  | reference |  |  | reference |  |  | reference |  |  |  |  |  |
| Single | -0.003 | 0.112 | 0.979 | -0.004 | 0.112 | 0.975 | 0.009 | 0.111 | 0.936 | <0.001 | 0.111 | 1.000 |  |  |  |
| Divorced/widowed/separated | 0.075 | 0.087 | 0.386 | 0.077 | 0.087 | 0.376 | 0.086 | 0.085 | 0.313 | 0.082 | 0.085 | 0.336 |  |  |  |
| **Race** |  |  |  |  |  |  |  |  |  |  |  |  |  |  |  |
| White | reference |  |  | reference |  |  | reference |  |  | reference |  |  |  |  |  |
| Black | 0.141 | 0.098 | 0.150 | 0.141 | 0.098 | 0.148 | 0.139 | 0.098 | 0.156 | 0.145 | 0.097 | 0.135 |  |  |  |
| Others | -0.268 | 0.140 | 0.055 | -0.266 | 0.140 | 0.057 | -0.265 | 0.139 | 0.058 | -0.264 | 0.139 | 0.059 |  |  |  |
| **Tumor size** |  |  |  |  |  |  |  |  |  |  |  |  |  |  |  |
| <2 cm | reference |  |  | reference |  |  | reference |  |  | reference |  |  | reference |  |  |
| 2-4 cm | 0.293 | 0.159 | 0.069 | 0.293 | 0.159 | 0.068 | 0.294 | 0.159 | 0.068 | 0.294 | 0.158 | 0.067 | 0.288 | 0.157 | 0.070 |
| ≥4 cm | 0.491 | 0.171 | 0.006 | 0.491 | 0.171 | 0.006 | 0.494 | 0.171 | 0.005 | 0.492 | 0.170 | 0.005 | 0.495 | 0.169 | 0.005 |
| **Pathology grade** |  |  |  |  |  |  |  |  |  |  |  |  |  |  |  |
| Well | reference |  |  | reference |  |  | reference |  |  |  |  |  |  |  |  |
| Moderately | -0.005 | 0.133 | 0.969 | -0.005 | 0.133 | 0.967 | 0.004 | 0.132 | 0.978 |  |  |  |  |  |  |
| Poorly/undifferetiated | 0.047 | 0.139 | 0.735 | 0.045 | 0.138 | 0.746 | 0.054 | 0.137 | 0.695 |  |  |  |  |  |  |
| **Radiotherapy** |  |  |  |  |  |  |  |  |  |  |  |  |  |  |  |
| None | reference |  |  | reference |  |  | reference |  |  | reference |  |  | reference |  |  |
| Beam | -0.450 | 0.098 | <0.001 | -0.449 | 0.098 | <0.001 | -0.443 | 0.098 | <0.001 | -0.441 | 0.097 | <0.001 | -0.440 | 0.097 | <0.001 |
| Beam+implants | -0.879 | 0.120 | <0.001 | -0.877 | 0.120 | <0.001 | -0.874 | 0.119 | <0.001 | -0.867 | 0.119 | <0.001 | -0.882 | 0.118 | <0.001 |
| Radiation, NOS | -0.291 | 0.201 | 0.149 | -0.294 | 0.201 | 0.144 | -0.293 | 0.201 | 0.146 | -0.292 | 0.201 | 0.147 | -0.299 | 0.200 | 0.135 |
| Implants | -1.001 | 0.184 | <0.001 | -1.000 | 0.184 | <0.001 | -0.991 | 0.184 | <0.001 | -0.988 | 0.183 | <0.001 | -0.985 | 0.183 | <0.001 |
| **Chemotherapy** |  |  |  |  |  |  |  |  |  |  |  |  |  |  |  |
| None/Unknown | reference |  |  | reference |  |  | reference |  |  | reference |  |  | reference |  |  |
| Yes | -0.303 | 0.084 | <0.001 | -0.303 | 0.084 | <0.001 | -0.302 | 0.084 | <0.001 | -0.305 | 0.084 | <0.001 | -0.296 | 0.084 | <0.001 |
| **Surgery** |  |  |  |  |  |  |  |  |  |  |  |  |  |  |  |
| None | reference |  |  | reference |  |  | reference |  |  | reference |  |  | reference |  |  |
| Local tumor excision | -0.570 | 0.124 | <0.001 | -0.575 | 0.124 | <0.001 | -0.569 | 0.124 | <0.001 | -0.567 | 0.124 | <0.001 | -0.551 | 0.123 | <0.001 |
| Vulvectomy | -0.737 | 0.144 | <0.001 | -0.742 | 0.143 | <0.001 | -0.745 | 0.143 | <0.001 | -0.748 | 0.143 | <0.001 | -0.739 | 0.142 | <0.001 |
| Debulking | -0.169 | 0.432 | 0.696 | -0.174 | 0.432 | 0.687 | -0.198 | 0.431 | 0.645 | -0.200 | 0.431 | 0.643 | -0.256 | 0.430 | 0.552 |
| **Number of lymph nodes removed** |  |  |  |  |  |  |  |  |  |  |  |  |  |  |  |
| None | reference |  |  | reference |  |  | reference |  |  | reference |  |  | reference |  |  |
| 1-3 | 0.208 | 0.272 | 0.445 | 0.208 | 0.273 | 0.446 | 0.219 | 0.272 | 0.421 | 0.225 | 0.271 | 0.408 | 0.194 | 0.272 | 0.476 |
| 4 or more | -0.424 | 0.182 | 0.020 | -0.420 | 0.182 | 0.021 | -0.438 | 0.181 | 0.016 | -0.441 | 0.181 | 0.015 | -0.455 | 0.181 | 0.012 |
| Number unknown | 0.387 | 0.425 | 0.362 | 0.393 | 0.425 | 0.354 | 0.395 | 0.425 | 0.353 | 0.404 | 0.424 | 0.341 | 0.366 | 0.424 | 0.389 |
| **T stage** |  |  |  |  |  |  |  |  |  |  |  |  |  |  |  |
| T1 | reference |  |  | reference |  |  | reference |  |  | reference |  |  | reference |  |  |
| T2 | 0.234 | 0.100 | 0.019 | 0.234 | 0.100 | 0.019 | 0.234 | 0.099 | 0.019 | 0.233 | 0.099 | 0.019 | 0.238 | 0.099 | 0.017 |
| T3 | 0.451 | 0.122 | <0.001 | 0.451 | 0.122 | <0.001 | 0.449 | 0.122 | <0.001 | 0.448 | 0.121 | <0.001 | 0.435 | 0.121 | <0.001 |
| T4 | 0.819 | 0.130 | <0.001 | 0.823 | 0.130 | <0.001 | 0.820 | 0.129 | <0.001 | 0.821 | 0.129 | <0.001 | 0.836 | 0.128 | <0.001 |
| TX | 0.113 | 0.148 | 0.446 | 0.111 | 0.148 | 0.454 | 0.100 | 0.148 | 0.497 | 0.109 | 0.148 | 0.462 | 0.133 | 0.147 | 0.365 |
| **N stage** |  |  |  |  |  |  |  |  |  |  |  |  |  |  |  |
| N0 | reference |  |  | reference |  |  | reference |  |  | reference |  |  | reference |  |  |
| N1 | 0.246 | 0.097 | 0.012 | 0.247 | 0.097 | 0.011 | 0.249 | 0.097 | 0.010 | 0.250 | 0.097 | 0.010 | 0.265 | 0.097 | 0.006 |
| NX | 0.275 | 0.138 | 0.046 | 0.277 | 0.138 | 0.044 | 0.280 | 0.137 | 0.042 | 0.279 | 0.137 | 0.042 | 0.276 | 0.137 | 0.044 |
| **M stage** |  |  |  |  |  |  |  |  |  |  |  |  |  |  |  |
| M0 | reference |  |  | reference |  |  | reference |  |  | reference |  |  | reference |  |  |
| M1 | 0.674 | 0.101 | <0.001 | 0.673 | 0.101 | <0.001 | 0.674 | 0.101 | <0.001 | 0.686 | 0.099 | <0.001 | 0.670 | 0.099 | <0.001 |
| MX | -0.105 | 0.231 | 0.650 | -0.104 | 0.231 | 0.654 | -0.102 | 0.231 | 0.659 | -0.107 | 0.228 | 0.639 | -0.127 | 0.227 | 0.577 |
| **Histology** |  |  |  |  |  |  |  |  |  |  |  |  |  |  |  |
| Squamous cell carcinoma | reference |  |  | reference |  |  |  |  |  |  |  |  |  |  |  |
| Adenocarcinoma | -0.082 | 0.101 | 0.414 | -0.083 | 0.101 | 0.410 |  |  |  |  |  |  |  |  |  |
| **Presence of other malignancies** |  |  |  |  |  |  |  |  |  |  |  |  |  |  |  |
| No | reference |  |  |  |  |  |  |  |  |  |  |  |  |  |  |
| Yes | -0.040 | 0.100 | 0.689 |  |  |  |  |  |  |  |  |  |  |  |  |
