# Supplementary material for: Development and validation of models for predicting the overall survival and cancer-specific survival of patients with primary vaginal cancer: A population-based retrospective cohort study
Source: Front Med (Lausanne). 2022 Aug 29;9:919150. doi: 10.3389/fmed.2022.919150 (PMC9464817; doi:10.3389/fmed.2022.919150)
Supplement: Supplementary file 3 [file Table_2.DOCX]

**Supplement Table 2| Results of the Candidate Multivariate Cox Proportional Hazard Models of Cancer-Specific Survival**

| **Varibles** | **Model 1** | | | **Model 2** | | | **Model 3** | | | **Model 4** | | | **Model 5** | | |
| --- | --- | --- | --- | --- | --- | --- | --- | --- | --- | --- | --- | --- | --- | --- | --- |
|  | **β** | **SE** | **p-value** | **β** | **SE** | **p-value** | **β** | **SE** | **p-value** | **β** | **SE** | **p-value** | **β** | **SE** | **p-value** |
| **Age** |  |  |  |  |  |  |  |  |  |  |  |  |  |  |  |
| 18-39 | reference |  |  | reference |  |  | reference |  |  | reference |  |  | reference |  |  |
| 40-59 | 0.096 | 0.270 | 0.722 | 0.105 | 0.270 | 0.698 | 0.088 | 0.268 | 0.742 | 0.085 | 0.268 | 0.751 | 0.086 | 0.266 | 0.747 |
| 60-79 | 0.386 | 0.267 | 0.147 | 0.391 | 0.266 | 0.142 | 0.379 | 0.266 | 0.154 | 0.377 | 0.266 | 0.156 | 0.368 | 0.262 | 0.161 |
| 80-100 | 1.137 | 0.279 | <0.001 | 1.150 | 0.279 | <0.001 | 1.137 | 0.278 | <0.001 | 1.135 | 0.278 | <0.001 | 1.111 | 0.270 | <0.001 |
| **Marital status** |  |  |  |  |  |  |  |  |  |  |  |  |  |  |  |
| Married | reference |  |  | reference |  |  | reference |  |  | reference |  |  |  |  |  |
| Single | -0.047 | 0.129 | 0.716 | -0.035 | 0.128 | 0.785 | -0.048 | 0.127 | 0.706 | -0.039 | 0.126 | 0.757 |  |  |  |
| Divorced/widowed/separated | -0.084 | 0.102 | 0.409 | -0.076 | 0.101 | 0.454 | -0.081 | 0.100 | 0.417 | -0.070 | 0.099 | 0.481 |  |  |  |
| **Race** |  |  |  |  |  |  |  |  |  |  |  |  |  |  |  |
| White | reference |  |  | reference |  |  | reference |  |  |  |  |  |  |  |  |
| Black | 0.038 | 0.118 | 0.745 | 0.036 | 0.118 | 0.763 | 0.040 | 0.117 | 0.732 |  |  |  |  |  |  |
| Others | -0.216 | 0.161 | 0.181 | -0.214 | 0.161 | 0.184 | -0.217 | 0.161 | 0.178 |  |  |  |  |  |  |
| **Tumor size** |  |  |  |  |  |  |  |  |  |  |  |  |  |  |  |
| <2 cm | reference |  |  | reference |  |  | reference |  |  | reference |  |  | reference |  |  |
| 2-4 cm | 0.304 | 0.203 | 0.138 | 0.304 | 0.203 | 0.139 | 0.304 | 0.202 | 0.135 | 0.296 | 0.201 | 0.145 | 0.296 | 0.202 | 0.145 |
| ≥4 cm | 0.612 | 0.218 | 0.007 | 0.614 | 0.218 | 0.007 | 0.613 | 0.216 | 0.006 | 0.609 | 0.214 | 0.006 | 0.605 | 0.214 | 0.006 |
| **Pathology grade** |  |  |  |  |  |  |  |  |  |  |  |  |  |  |  |
| Well | reference |  |  | reference |  |  |  |  |  |  |  |  |  |  |  |
| Moderately | -0.067 | 0.161 | 0.680 | -0.056 | 0.159 | 0.727 |  |  |  |  |  |  |  |  |  |
| Poorly/undifferetiated | 0.024 | 0.164 | 0.883 | 0.034 | 0.163 | 0.833 |  |  |  |  |  |  |  |  |  |
| **Radiotherapy** |  |  |  |  |  |  |  |  |  |  |  |  |  |  |  |
| None | reference |  |  | reference |  |  | reference |  |  | reference |  |  | reference |  |  |
| Beam | -0.501 | 0.115 | <0.001 | -0.495 | 0.114 | <0.001 | -0.493 | 0.114 | <0.001 | -0.491 | 0.114 | <0.001 | -0.492 | 0.114 | <0.001 |
| Beam+implants | -1.073 | 0.145 | <0.001 | -1.069 | 0.145 | <0.001 | -1.061 | 0.144 | <0.001 | -1.062 | 0.144 | <0.001 | -1.057 | 0.144 | <0.001 |
| Radiation, NOS | -0.267 | 0.230 | 0.245 | -0.267 | 0.230 | 0.245 | -0.264 | 0.229 | 0.250 | -0.252 | 0.229 | 0.271 | -0.245 | 0.228 | 0.284 |
| Implants | -1.186 | 0.233 | <0.001 | -1.177 | 0.233 | <0.001 | -1.169 | 0.233 | <0.001 | -1.161 | 0.233 | <0.001 | -1.161 | 0.233 | <0.001 |
| **Chemotherapy** |  |  |  |  |  |  |  |  |  |  |  |  |  |  |  |
| None/Unknown | reference |  |  | reference |  |  | reference |  |  | reference |  |  | reference |  |  |
| Yes | -0.219 | 0.101 | 0.029 | -0.219 | 0.100 | 0.030 | -0.223 | 0.100 | 0.026 | -0.215 | 0.100 | 0.032 | -0.211 | 0.100 | 0.034 |
| **Surgery** |  |  |  |  |  |  |  |  |  |  |  |  |  |  |  |
| None | reference |  |  | reference |  |  | reference |  |  | reference |  |  | reference |  |  |
| Local tumor excision | -0.632 | 0.160 | <0.001 | -0.626 | 0.160 | <0.001 | -0.624 | 0.160 | <0.001 | -0.611 | 0.160 | <0.001 | -0.611 | 0.160 | <0.001 |
| Vulvectomy | -0.694 | 0.174 | <0.001 | -0.698 | 0.174 | <0.001 | -0.699 | 0.173 | <0.001 | -0.687 | 0.172 | <0.001 | -0.686 | 0.172 | <0.001 |
| Debulking | -0.063 | 0.438 | 0.886 | -0.091 | 0.437 | 0.835 | -0.093 | 0.436 | 0.832 | -0.098 | 0.437 | 0.822 | -0.074 | 0.435 | 0.866 |
| **Number of lymph nodes removed** |  |  |  |  |  |  |  |  |  |  |  |  |  |  |  |
| None | reference |  |  | reference |  |  | reference |  |  | reference |  |  | reference |  |  |
| 1-3 | 0.178 | 0.315 | 0.572 | 0.189 | 0.314 | 0.548 | 0.195 | 0.313 | 0.534 | 0.182 | 0.314 | 0.563 | 0.194 | 0.313 | 0.536 |
| 4 or more | -0.306 | 0.208 | 0.142 | -0.324 | 0.207 | 0.117 | -0.331 | 0.207 | 0.109 | -0.336 | 0.207 | 0.105 | -0.330 | 0.207 | 0.111 |
| Number unknown | 0.657 | 0.433 | 0.129 | 0.658 | 0.433 | 0.129 | 0.674 | 0.432 | 0.119 | 0.655 | 0.431 | 0.129 | 0.647 | 0.431 | 0.133 |
| **T stage** |  |  |  |  |  |  |  |  |  |  |  |  |  |  |  |
| T1 | reference |  |  | reference |  |  | reference |  |  | reference |  |  | reference |  |  |
| T2 | 0.374 | 0.123 | 0.002 | 0.374 | 0.123 | 0.002 | 0.373 | 0.123 | 0.002 | 0.382 | 0.123 | 0.002 | 0.383 | 0.123 | 0.002 |
| T3 | 0.642 | 0.145 | <0.001 | 0.640 | 0.145 | <0.001 | 0.636 | 0.145 | <0.001 | 0.641 | 0.144 | <0.001 | 0.643 | 0.144 | <0.001 |
| T4 | 0.998 | 0.149 | <0.001 | 0.995 | 0.149 | <0.001 | 1.001 | 0.148 | <0.001 | 1.016 | 0.147 | <0.001 | 1.017 | 0.147 | <0.001 |
| TX | 0.274 | 0.179 | 0.126 | 0.263 | 0.179 | 0.141 | 0.274 | 0.179 | 0.125 | 0.288 | 0.178 | 0.106 | 0.288 | 0.178 | 0.104 |
| **N stage** |  |  |  |  |  |  |  |  |  |  |  |  |  |  |  |
| N0 | reference |  |  | reference |  |  | reference |  |  | reference |  |  | reference |  |  |
| N1 | 0.222 | 0.110 | 0.043 | 0.226 | 0.110 | 0.040 | 0.228 | 0.110 | 0.038 | 0.237 | 0.109 | 0.030 | 0.240 | 0.109 | 0.028 |
| NX | 0.097 | 0.170 | 0.567 | 0.100 | 0.169 | 0.553 | 0.098 | 0.169 | 0.561 | 0.098 | 0.169 | 0.562 | 0.096 | 0.169 | 0.570 |
| **M stage** |  |  |  |  |  |  |  |  |  |  |  |  |  |  |  |
| M0 | reference |  |  | reference |  |  | reference |  |  | reference |  |  | reference |  |  |
| M1 | 0.707 | 0.113 | <0.001 | 0.707 | 0.113 | <0.001 | 0.723 | 0.111 | <0.001 | 0.715 | 0.110 | <0.001 | 0.716 | 0.110 | <0.001 |
| MX | 0.200 | 0.264 | 0.450 | 0.201 | 0.265 | 0.450 | 0.201 | 0.263 | 0.445 | 0.192 | 0.262 | 0.464 | 0.194 | 0.262 | 0.460 |
| **Presence of other malignancies** |  |  |  |  |  |  |  |  |  |  |  |  |  |  |  |
| No | reference |  |  | reference |  |  | reference |  |  | reference |  |  | reference |  |  |
| Yes | -0.510 | 0.142 | <0.001 | -0.513 | 0.142 | <0.001 | -0.505 | 0.142 | <0.001 | -0.497 | 0.142 | <0.001 | -0.490 | 0.141 | 0.001 |
| **Histology** |  |  |  |  |  |  |  |  |  |  |  |  |  |  |  |
| Squamous cell carcinoma | reference |  |  |  |  |  |  |  |  |  |  |  |  |  |  |
| Adenocarcinoma | -0.083 | 0.118 | 0.482 |  |  |  |  |  |  |  |  |  |  |  |  |
